# Genie in the bottle? a qualitative study of general practitioners’ perspectives and information needs concerning digital mental health applications in Germany

**DOI:** 10.1186/s12875-025-03115-2

**Published:** 2025-12-09

**Authors:** Fatma Sahan, Karin Panitz, Charlotte Wagenaar, Nadja Kairies-Schwarz, Markus Vomhof, Maximilian Zinn, Lisa Guthardt, Jessica Bau, Adrian Loerbroks, Claudia R. Pischke, Jennifer Apolinário-Hagen

**Affiliations:** 1 Institute for Occupational, Social and Environmental Medicine, Center for Health and Society, Faculty of Medicine, Heinrich Heine University Düsseldorf and University Hospital Düsseldorf, Düsseldorf, Germany; 2 Center for Digital Medicine, Faculty of Medicine, Heinrich Heine University Düsseldorf and University Hospital Düsseldorf, Düsseldorf, Germany; 3 Institute for Health Services Research and Health Economics, Medical Faculty, Center for Health and Society, University Hospital Düsseldorf, Heinrich Heine University Düsseldorf, Düsseldorf, Germany; 4 Institute for Health Services Research and Health Economics, German Diabetes Center, Leibniz Center for Diabetes Research at the Heinrich Heine University Düsseldorf, Düsseldorf, Germany; 5 Institute of Medical Sociology, Medical Faculty and University Hospital Düsseldorf, Center for Health and Society, Heinrich Heine University Düsseldorf, Düsseldorf, Germany

**Keywords:** Physicians, General practitioners, Digital health, Mental health, Telemedicine, Mobile applications, Mental health services, Qualitative research, Information literacy

## Abstract

**Background:**

The concerning prevalence of mental disorders underscores the need for innovative solutions in outpatient care, including prescription digital therapeutics (DTx) designed as regulated software-based medical products for treatment and disease management. In Germany, approved DTx are called DiGAs (referring to “Digitale Gesundheitsanwendungen”) and can be prescribed since 2020 at the expense of statutory health insurance. However, DiGAs remain underutilized by general practitioners (GPs) in primary care. Although prior research indicated individual barriers to prescription among healthcare professionals, little is known on how acceptance-facilitating strategies could be tailored to GPs’ needs. This study therefore explores GPs’ perspectives on DiGAs in general and for mental health, focusing on their needs and wishes regarding information strategies.

**Methods:**

A qualitative descriptive exploratory study using semi-structured interviews with GPs was conducted within a mixed-methods study in fall 2024. Participants were recruited via GP networks, social media, and fax using a purposive sampling approach. Data was analyzed using qualitative content analysis with deductive-inductive category development.

**Results:**

Thirteen GPs between 27 and 66 years (median: 54 years, female: *n* = 3; 23%) were interviewed. Twelve participants (92%) had prescribed DiGAs at least once, mostly based on patients’ requests for dealing with depression, insomnia or obesity. Analysis revealed varying levels of perceived knowledge and differing attitudes toward digitalization. Independent information sources, particularly from governmental and regulatory institutions, medical associations, and colleagues were mentioned, while ambivalent views on health insurances were reported. GPs favored information content on the evidence base, indications, usability, and cost effectiveness of DiGAs. In terms of delivery modes, online formats, in-person events, and traditional print media were named.

**Conclusions:**

GPs showed high engagement with DiGA prescriptions despite persistent knowledge gaps and ambivalent attitudes. They expressed expectations regarding information provision, emphasizing concise content from trusted, neutral sources, about central structural aspects (e.g., budgetary impact), delivered through familiar formats such as journals, lectures, and digital platforms. Information strategies should account for limited time resources in primary care and align with established routines. Strengthening institutional support and integrating digital health into medical training may further facilitate the adoption of DiGAs in primary care.

**Supplementary Information:**

The online version contains supplementary material available at 10.1186/s12875-025-03115-2.

## Introduction

The prevalence of common mental disorders (CMDs), including depression and anxiety, burdens the healthcare systems worldwide and has increased significantly [1–3]. Digital therapeutics (DTx), including digital Mental Health Interventions (dMHI), have the potential to serve as valuable resources for patients and the entire outpatient healthcare system. As gatekeepers in primary care, general practitioners (GPs) play a pivotal role in identifying suitable patients, initiating treatment pathways, and integrating DTx into routine care for individuals with mental health issues.

**Fig. 1:**
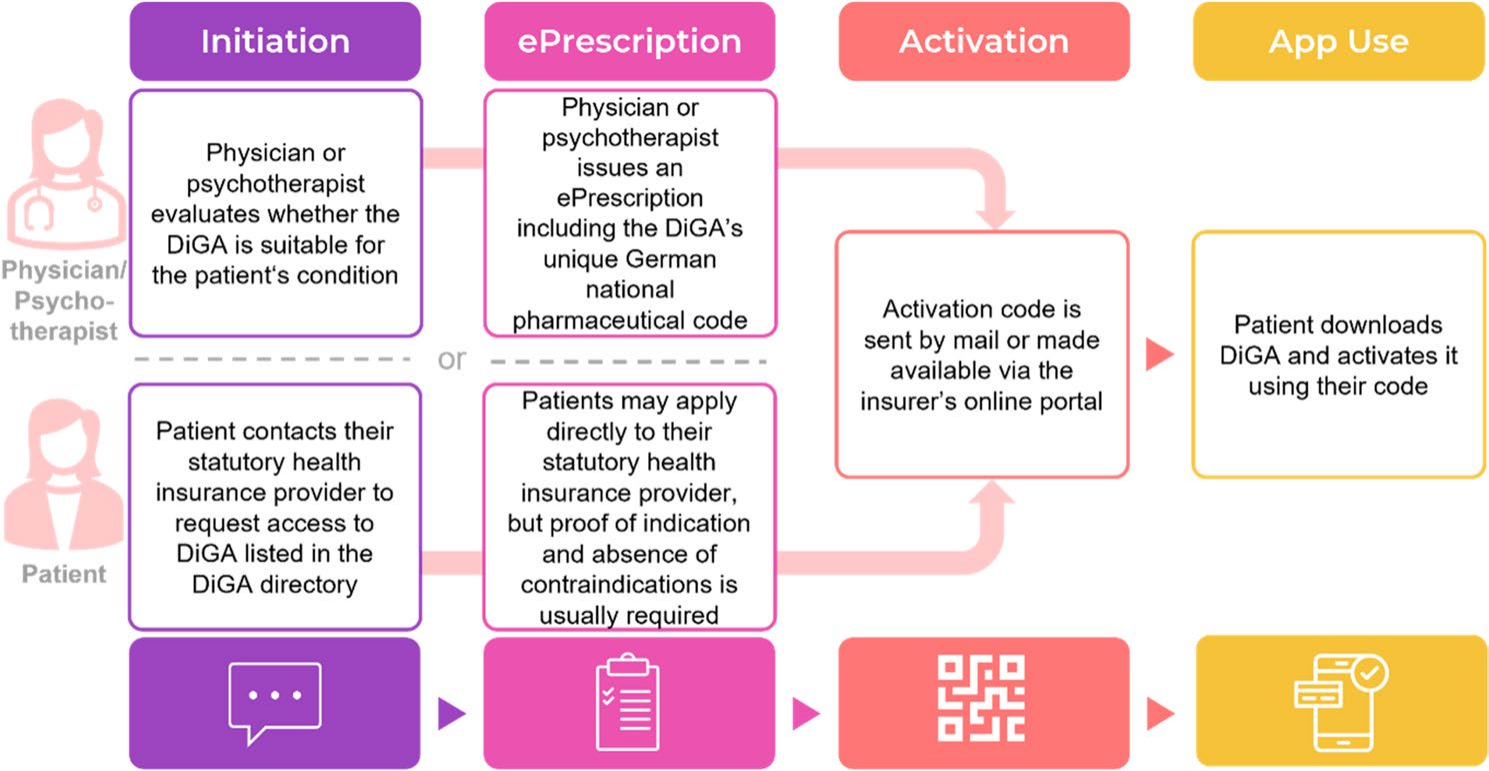
Pathways to access to digital health applications (DiGAs) in Germany via physician or psychotherapist prescription versus health insurance application. Abbreviations: DiGAs = Digital health applications (German “Digitale Gesundheitsanwendungen“, i.e., prescription digital therapeutics). This flow diagram illustrates the two official access pathways to receive access to DiGAs in Germany (via conventional prescription versus direct request to the statutory health insurance provider). In both cases the patient receives a time-limited activation code (usually valid for 90 days) and downloads the DiGA from an app store. A physician prescription is not mandatory, as patients may also apply directly to their insurer, but proof of indication and absence of contraindications is usually required [10, 11]. Since October 2024, physicians and psychological psychotherapists in Germany are only allowed to prescribe DiGAs if their practice management software is certified by the National Association of Statutory Health Insurance Physicians. Figure created by the authors, adapted from information provided by the Federal Ministry of Health [12]

In several European healthcare systems, the implementation of DTx has only recently been initiated or completed [4]. In this context, Germany has taken a pioneering role with the implementation of digital health applications (Digitale Gesundheitsanwendungen, DiGAs), commonly referred to as “apps on prescription” [5]. DiGAs are software-based Conformité Européenne (CE)-certified medical products approved for the detection, monitoring, treatment, or alleviation of a broad range of diseases, including mental health conditions [6–9]. The legal basis for DiGAs was founded with the passing of the Digital Healthcare Act (Digitale-Versorgung-Gesetz, DVG) in December 2019, which led to the establishment of the DiGA directory by the Federal Institute for Drugs and Medical Devices (Bundesinstitut für Arzneimittel und Medizinprodukte, BfArM), where applications can be listed either permanently or on a provisional basis pending evidence of benefit [6, 7]. Since fall of 2020, DiGAs can be prescribed for patients with statutory health insurance in Germany, with costs reimbursed by the insurers. In addition to prescription by physicians or psychotherapists, patients may also obtain access by requesting coverage from their statutory health insurance [8]. An overview of the available access pathways to DiGAs is illustrated in Fig. 1.

For patients with mental health issues, GPs often serve as the first point of contact or a compensatory resource given the limited access to psychotherapy [13]. DiGAs may support patient care by enhancing care quality and efficiency while reducing healthcare costs [14]. Especially in rural settings, they may help improve access and reduce travel and waiting times [15, 16].

Meta-analyses have confirmed the efficacy of both therapist- and self-guided dMHI based on cognitive behavioral therapy (CBT), particularly for CMDs [17–19]. However, the uptake of DiGAs remains modest [9]. A survey conducted in 2021 by Dahlhausen et al. [20] revealed that less than 10% of surveyed physicians and psychotherapists in Germany (*N* = 1299) have ever prescribed any DiGA, while around a third of them plan to prescribe one within the next year. Although DiGAs are increasingly recognized by internists for their potential to enhance patient care [21], actual use remains low. As of June 2023, only 12% of the approximately 185.000 physicians and psychotherapists participating in outpatient statutory care had issued a DiGA prescription to insured patients [22].

The barriers to the prescription of DiGAs are multifactorial. Structural challenges such as time constraints, high workload [23], outdated IT infrastructure, and the resulting difficulties in integrating DiGAs into existing work processes are cited [21, 24, 25]. In addition, uncertainties regarding specific DiGA related aspects, such as the prescription process, reimbursement regulations, and concerns about patient adherence persist [21, 24, 25]. Studies have shown that medical professionals report insufficient knowledge about DiGAs, expressing issues with finding reliable, easily accessible information in case of need [26, 27]. However, little is known about how information on DiGAs should be designed and delivered to meet the needs of GPs and encourage their practical use in primary care.

### The role of information in gps’ adoption of DiGAs

The integration of DTx into routine care requires physicians to overcome challenges like becoming familiar with novel and changing regulations and adapting workflows, demanding time and reliable information on various aspects, such as effectiveness and patient suitability [28]. Research indicates that greater perceived knowledge about DiGAs is positively associated with the frequency of prescriptions [21], whereas insufficient information can foster skepticism toward their use [24]. Despite the increased accessibility of digital information resources in contemporary medicine, studies show that physicians primarily rely on memory for information retention [29]. Given the continuous proliferation of medical knowledge, which is estimated to double every 20 years [30, 31], maintaining up-to-date knowledge in routine clinical practice can be difficult. In addition to attitudes toward new technologies [32, 33], structural challenges such as demographic shifts [34], shortages of GPs in rural regions [35], and gaps in the digital infrastructure of the healthcare system [36] further complicate the integration of DiGAs. As a result, many clinical questions in everyday practice, such as diagnostic or therapeutic uncertainties, remain unanswered [37], which may impair the quality of medical decisions and treatment outcomes [38], and reduce the willingness to prescribe DiGAs. In their research information, physicians less frequently use digital evidence based information resources [39]. Conversely, they continue to favor traditional media such as journals, textbooks, guidelines, continuing medical education (CME), conferences, and peer-to-peer exchange [39]. These preferred information channels are only suitable for communicating innovative treatments such as DiGAs to a limited extent. The underutilization of digital information sources complicates the ongoing engagement with the rapidly evolving range of digital health solutions, thereby constraining the capacity for evidence-based evaluation of novel applications. Unmet information needs may thus contribute to hesitant prescription of DiGAs.

### Artificial intelligence as digital innovation in healthcare

While DiGAs are currently the most prominent example of DTx in Germany, they are part of a broader spectrum of digital innovations in healthcare. Some DiGAs already incorporate artificial intelligence (AI)-based elements [40], and its integration is projected to increase in the near future [41]. In the context of DTx, AI can help to make diagnoses earlier, faster, and more accurate; simplify and optimize processes such as scheduling and documentation; support the relationship between doctors and patients by providing personalized recommendations; and save time and resources [42]. It is therefore important to investigate GPs’ attitudes toward AI alongside their views on DiGAs, in order to capture their perceptions of digital innovations in healthcare at an early stage of their implementation into routine medical practice.

### Aim of the study

This study aims to explore GPs’ perspectives on their information needs and preferences concerning dMHI, and particularly DiGAs, in the field of mental health. While existing literature has indicated that physicians experience a general lack of information, little is known about how information strategies should be designed to effectively meet their needs. A qualitative approach was therefore deemed appropriate, as it allows for an in-depth exploration of GPs’ views on who should provide information about DiGAs, which content should be addressed, and through which delivery modes. The findings aim to enhance information dissemination and thereby foster the integration of digital applications into the healthcare system.

### Research Questions (RQ):

1. How do GPs perceive their current level of knowledge and experience regarding DiGAs?
2. How are GPs’ attitudes toward patient-centered digitalization in medical practice, including the diffusion of DiGAs and AI into primary care?
3. Whose initiative leads to the prescription of DiGAs in general practice?
4. From which sources do GPs wish to receive information about DiGAs?
5. What type of content do GPs consider most relevant when learning about DiGAs?
6. Through which delivery modes do GPs want to receive information about DiGAs?
7. How much time are GPs willing to invest in learning about DiGAs?

## Methods

This qualitative study was conducted as part of a mixed-methods research project with different groups of healthcare professionals (HCPs), including GPs, occupational physicians and psychotherapists, to shed light on the diverse perspectives on underexplored information needs and preferences regarding DiGAs for mental disorders. Prior to data collection, the qualitative study was approved by the Ethics Committee of the Medical Faculty of Heinrich Heine University Düsseldorf (HHU; study number: 2023–2338). The reporting follows Consolidated Criteria for Reporting Qualitative Research (COREQ, see Supplementary Table 1; [43]).

### Research team

The research was conducted by a multidisciplinary team. JAH (psychologist, postdoctoral qualification in health sciences) has expertise in basic research in digital health acceptance and served as principal investigator (PI), in collaboration with NKS (professor of behavioral health economics). Together with members of NKS’ research group, MV and MZ (economists specialized in preference research in primary care), they supported the preparatory work for this study. FS, a doctoral candidate in Public Health with a background in experimental psychology and neuroscience, was primarily responsible for data analysis. She collaborated closely with the PIs and received analytical feedback from LG and JB, both doctoral researchers with expertise in qualitative methods. Interviews were conducted by KP and CW, both doctoral researchers in Public Health and Medicine. KP performed a related sub-study on psychotherapists’ DiGA information needs [44]. FS, KP, and CW received in-depth training in qualitative methods and analysis. AL, a health scientist with expertise in health service research, provided methodological guidance. All team members contributed to the design, analysis, or interpretation in line with their disciplinary backgrounds in psychology (FS, KP, CP, JAH), medicine (CW), health economics (NKS, MV, MZ), public health (LG, JB, AL, CP), and occupational medicine (AL, JAH). The research team’s interest in studying the acceptance of DiGAs arose from a broader engagement with digital health research, especially drivers and barriers to implementation from the HCPs’ perspective and acceptance-facilitating interventions [45–47]. None of the researchers were involved in the commercial distribution of DiGAs.

### Sampling strategy

A purposive sampling strategy was used to recruit GPs with specialist certification in internal or general medicine, practical experience in primary care, and authorization to prescribe DiGAs in Germany. Eligibility criteria further included written informed consent, active clinical practice in Germany, proficiency in German, and either statutory health insurance accreditation or employment at an institution authorized to prescribe DiGAs.

Recruitment was conducted via multiple channels: social media platforms (e.g., Instagram and LinkedIn), using flyers and short informational videos; local academic and clinical networks of the Institute of General Practice (Institut für Allgemeinmedizin, IfAM) and Primary Care Research Practice Network (Hausärztliches Forschungspraxennetzwerk, HAFO) at HHU and its University Hospital; the mailing list of the Occupational Medicine Network of Ludwig Maximilian University Munich (ArbMedNet); targeted fax invitations to 50 GPs identified via the publicly available physician directory [48]; and personal healthcare contacts of the research team. Data on response rates were not available, as recruitment was based on mailing lists and flyer distribution, with interested physicians contacting the research team directly. No participant dropped out.

The sample also included physicians with dual qualifications in general medicine and occupational medicine, as well as one resident in general practice. In the German healthcare system, it is common for GPs to additionally hold certification in occupational medicine. As these physicians primarily provide general medical care in a primary care setting, they were regarded as part of the primary care group. Recruitment continued until data and meaning saturation was achieved, indicating that no substantially new themes emerged during the final interviews.

### Study design and procedure

Before participating, the recruited GPs received written study information, a consent form, and a short questionnaire capturing sociodemographic (e.g., age, gender, professional experience, federal state) and professional data (e.g., DiGA experience, employment type; see Supplementary Table 2).

The study followed a qualitative research design using semi-structured interviews to explore GPs‘ information needs regarding DiGAs for mental health issues. The interview guide (see Supplementary Table 3) covered four thematic blocks: (1) perceived prior knowledge about DiGAs; (2) attitudes toward digitalization in medical practice; (3) experiences with DiGA use or prescription; (4) information needs regarding information sources, contents, and delivery modes when gathering information about DiGAs. The guide was developed grounded in empirical research [45–47] and iteratively refined based on internal feedback. To ensure clarity and methodological quality, four pretest interviews were carried out with a doctoral student, a medical student, a junior physician, and a GP. Minor linguistic refinements were made based on this feedback. Interviews were conducted online from August 23 until October 25, 2024 using the general data protection regulations (GDPR)-compliant videoconferencing software CISCO Webex (California, USA [49]), with access provided by the institution. Participants were informed of their option to decline the webcam usage. The interviewers introduced themselves as a psychologist (KP) and a medical graduate (CW) prior to the commencement of the interview, providing a concise explanation of their personal interest in dMHI. During the online interview, only the participant and the interviewer were present. The video was not recorded. Rather, audio files were obtained using a GDPR-compliant voice recorder (Olympus). In total, 13 GPs were interviewed. Except for one participant who had a personal relationship with KP, no prior relationship existed between the researchers and the study participants. All participants received a reimbursement of €100 for completing the respective interview.

### Data analysis

The transcription and qualitative content analysis of the interviews was conducted using MAXQDA 2024 software in the version 24.8.0 [50]. The interviews were audio-recorded, pseudonymized, and transcribed verbatim using MAXQDA-embedded AI assistance with subsequent manual control, ensuring GDPR compliance. The transcriptions were subjected to a meticulous review process, during which they were revised and checked for accuracy by the respective interviewers.

The qualitative content analysis followed the methodological approach of Kuckartz and Rädiker [51]. Initial coding of the transcripts was performed by FS using a category system whose main categories were developed deductively based on the interview guide. This system was subsequently refined through the inductive development of subcategories grounded in the interview material. Preliminary findings were reviewed with JAH, LG, and JB. Based on their feedback, the category system was revised, and FS conducted a second phase of coding. During this phase, existing categories were further differentiated, and additional categories were introduced where needed to adequately capture the diversity of the content. The coding scheme continued to evolve through an iterative process, with deliberate sessions held to assess clarity, completeness, and parsimony. The final coding system, including anchor examples, is presented in Supplementary Table 4. All quotes included in the manuscript were translated from German into English by FS. To ensure accuracy and preserve meaning, LG cross-checked and verified the translations.

## Results

### Sample characteristics

A total of 13 GPs participated in the study, with the interviews lasting between 33:40 min and 51:40 min. The age of the interviewees ranged from 27 to 66 years, with an average age of 49 years (*M* = 48.92, *SD* = 14.63, median = 54 years). The majority of participants were male (*n* = 10; 76.92%), worked in North Rhine-Westphalia (*n* = 11; 84.62%), and in an urban area (*n* = 8; 61.54%). Most had a statutory health insurance license (*n* = 9; 69.23%), worked full-time (*n* = 12; 92.31%), and had a mean professional experience of 20 years (*M* = 20.08, *SD* = 12.55). Female GPs (*n* = 3) were on younger average (median = 34 years) than male GPs (median = 54). Most of the GPs surveyed have already prescribed a DiGA (*n* = 12; 92.31%), primarily targeting obesity (*n* = 10), depression (*n* = 9), sleep disorders (*n* = 3), anxiety disorders (*n* = 2), smoking cessation (*n* = 2), diabetes (*n* = 1), migraine (*n* = 1), panic disorders (*n* = 1), and tinnitus (*n* = 1). Of the 13 participating physicians, 10 were working exclusively in primary care practices, while three combined primary care with additional roles in occupational or outpatient clinical settings. As illustrated in Table 1, descriptive data is presented according to gender.

**Table 1:** Descriptive data according to gender

	Total (*N* = 13)	Men (*n* = 10)	Women (*n* = 3)
Age (years)			
*M (SD)*	48.92 (14.63)	53.4 (13.68)	34.0 (2.65)
Median	54	58	34
Professional experience (years)			
*M (SD)*	20.08 (12.55)	23.50 (12.38)	8.67 (1.53)
Median	20	24	9
Work area			
Rural (%)	5 (38.46)	3 (30.00)	2 (66.67)
Urban (%)	8 (61.54)	7 (70.00)	1 (33.33)
Health insurance license			
Yes (%)	9 (69.23)	8 (80.00)	1 (33.33)
No (%)	4 (30.80)	2 (20.00)	2 (66.67)
Employment type			
Full-time (%)	12 (92.31)	9 (90.00)	3 (100.00)
Part-time (%)	1 (7.69)	1 (10.00)	0
Prescription of DiGAs in the past			
Yes (%)	12 (92.31)	9 (90.00)	3 (100.00)
No (%)	1 (7.69)	1 (10.00)	0

*Notes.* Abbreviations: DiGAs = Digital health applications (German “Digitale Gesundheitsanwendungen”, i.e., prescription of digital therapeutics). It should be noted that DiGAs can be prescribed not only by office-based physicians with a statutory health insurance license, but also by physicians working in institutional or outpatient settings without an accreditation

### Qualitative main results

We identified seven main categories from the qualitative data: [1] perceived level of knowledge of and experience with DiGAs; [2] attitudes toward digitalization in medical practice; [3] initiation of DiGA prescriptions; [4] needs and wishes regarding information sources; [5] needs and wishes regarding information content; [6] needs and wishes regarding information delivery modes; and [7] time investment related to DiGAs. Each of these categories was further divided into specific subcategories.

#### Prior knowledge of and experience with DiGAs (RQ 1: How do GPs perceive their current level of knowledge and experience regarding DiGAs?)

To assess the perceived need for information, GPs were asked to indicate their level of knowledge about DiGAs. Some participants described their knowledge as limited, noting that DiGAs had not yet been implemented in their practice. Others stated a high level of knowledge, articulating detailed insights into the regulatory status, therapeutic orientation, and applicability of specific DiGAs.

*“There are*,* of course*,* those very specifically*,* predominantly behavior therapy-oriented apps that do quite a lot themselves*,* interacting with the patients beyond psychoeducation. And this becomes relevant when patients are familiar with the electronic devices required and when they’re willing to use them. And when the app is at least on the official list of the Federal Institute for Drugs and Medical Devices. There are different stages of approval*,* and for me it’s important that they are permanently listed products.”* (*GP13*,* male*).

#### Attitudes toward digitalization in medical practice (RQ2: How are GPs’ attitudes toward patient-centered digitalization in medical practice, including the diffusion of DiGAs and AI into primary care?)

GPs had different attitudes about the digitalization of medical practice, including DiGAs and AI. While some highlighted potential benefits, others voiced reservations and concerns.

### 2a Attitudes toward DiGAs

Participants noted the potential of DiGAs to improve access to psychotherapy, especially in underserved areas. DiGAs were also seen as a helpful tool for bridging the waiting period until a therapy slot becomes available. Furthermore, the 24/7 availability of the applications was considered beneficial, allowing patients to engage with the content flexibly, outside of practice hours. Another commonly stated benefit was the potential of DiGAs to compensate workforce shortages in healthcare by supporting therapeutic processes, as human resources are limited. There was a general openness among GPs toward prescribing DiGAs, especially for mental health-related indications.

*“Especially in the field of mental health*,* I consider DiGAs very useful. Many patients have urgent needs*,* but there is just a lack of human or physical capacity to take care of it. As general practitioners we are also bound by strict regulations and time constraints in what we can offer. DiGAs can be used by patients at any time. That’s why I would say I’m generally positive toward it.” (GP01*,* male).*

Some respondents with an indecisive stance acknowledged the potential of DiGAs but expressed a lack of enthusiasm for their implementation in their own practice. While they did not question the general value of digital applications, they tended to prioritize other areas of care or innovation, such as AI (see, section 2b). According to several respondents, the rapid and constant flow of medical innovations contributed to the perception that DiGAs are not an immediate priority. Some described their perspective as open but reserved, indicating that DiGAs might be useful in theory, yet not sufficiently compelling or relevant for their current clinical context.

Participants who expressed a negative attitude toward DiGAs raised concerns about the loss of direct contact between patients and HCPs. They feared that replacing or supplementing therapy with digital tools could compromise the patient-physician relationship.

*“What makes DiGA unattractive to me is the fact that it shifts everything to an impersonal*,* ultimately AI-based level*,* where personal exchange*,* direct communication*,* and the emotional dimension between people no longer take place.”* (*GP07*,* male*).

In this context, GPs reported several challenges related to prescribing DiGAs. They criticized the administrative burden, describing the process as too time-consuming relative to its perceived benefit. In addition, they highlighted the lack of technical integration of DiGAs in existing practice software and expressed a need for clear guidance on how to issue prescriptions. Another frequently mentioned concern was that older patients, who are often less familiar or comfortable with digital technologies, might struggle to use such applications effectively.

Furthermore, GPs emphasized the overwhelming volume of information in their daily routines, which can make it difficult to identify and evaluate new digital tools.

### 2b Attitudes toward Artificial Intelligence (AI)

Some participants expressed a favorable view of AI applications, particularly regarding their potential to reduce workloads in diagnostics or administrative tasks.

*“I am very much in favor of AI*,* particularly when it helps reduce the workload. This includes diagnostic support*,* of course*,* but also administrative tasks such as billing. Any form of relief that does not require additional staff would be highly appreciated.”* (*GP11*,* female*).

One interviewee also highlighted the potential of data-driven AI models to support medical decision-making, for example by comparing a large number of findings with a current patient case, even though they had not yet used such tools themselves.

Others expressed ambivalence toward AI. While there was some acceptance of technological progress, the current added value of AI in general practice was questioned.

*“At the moment*,* I don’t see any advantage of AI*,* except maybe for radiology findings*,* standard findings.”* (*GP04*,* male*).

It was also noted that, given the existing structural challenges in the healthcare system, other issues are currently seen as more pressing.

Some participants voiced skepticism or expressed generally negative attitudes toward the use of AI in medicine, emphasizing ethical concerns and a fundamental unease with new technology.

*“I would say that I am basically also rather critical toward AI*,* and I have quite some respects for the development and the use of these technologies*,* so I don’t think that keeping medical staff informed about every latest innovation in this field should be a top priority.”* (*GP07*,* male*).

### Initiators of DiGA prescription (RQ 3: Whose initiative leads to the prescription of DiGAs in general practice?)

DiGA prescriptions were initiated in different ways. GP-driven prescriptions reflected a more proactive approach, particularly in the context of mental health care. In contrast, some GPs stated that prescriptions were often based on patient requests.

However, it was also evident that a few GPs did not consistently respond positively to patient-initiated prescriptions, particularly in cases involving external service providers.

*“I received several faxes from a DiGA provider*,* including the name of my patients and asking me to return the completed form or prescription. I think that is presumptuous and intrusive.”* (*GP06*,* male*).

### Information-related needs and wishes among general practitioners

#### Perspectives regarding information sources (RQ 4: From which sources do GPs wish to receive information about DiGAs?)

GPs indicated a preference for accessing a variety of sources when gathering information about DiGAs. A structured categorization of these sources was developed on institutional affiliation, practical relevance, and perceived credibility. This typology aligned with the framework of the Federal Ministry of Health (Bundesministerium für Gesundheit, BMG [52]).

##### Governmental and regulatory bodies

Most participants expressed a clear interest in receiving information about DiGAs from central and authoritative institutions within the German healthcare system. Among the most frequently mentioned were the BfArM and the BMG. GPs also named the Institute for Quality and Efficiency in Health Care (Institut für Qualität und Wirtschaftlichkeit im Gesundheitswesen, IQWiG). These institutions were perceived as trustworthy providers of up-to-date, evidence-based, and industry-independent information.

In addition, several GPs highlighted the role of regional medical associations (Ärztekammer) and the National Association of Statutory Health Insurance Physicians (Kassenärztliche Bundesvereinigung) as trustworthy sources for information regarding DiGAs. They emphasized that such institutions are responsible for regulatory decisions and are therefore better positioned than commercial providers to deliver reliable, centralized information.

*“I think that*,* if anything*,* information should come from the Association of Statutory Health Insurance Physicians*,* since they decide what can be prescribed. The information should be centralized*,* I don’t care whether this is through the medical associations or the regional insurance physicians’ associations*,* but ideally*,* through a separate website.”* (*GP02*,* male*).

##### Professional associations

In addition, several GPs considered professional associations as reliable sources of information regarding DiGAs. Among the organizations mentioned were the German Association of General Practitioners (Deutscher Hausärzteverband) and the German Society for General Practice and Family Medicine (Deutsche Gesellschaft für Allgemeinmedizin und Familienmedizin; DEGAM).

One GP emphasized the importance of scientific evidence and expressed the need for “guideline-like recommendations” for DiGAs (*GP10*,* male*). He underlined that such recommendations, similar to clinical guidelines, should remain non-binding to preserve physicians’ freedom of choice.

##### Statutory health insurances

Some GPs were interested in receiving information on DiGAs from statutory health insurances who represent the funding bodies. Others adopted a more critical stance, questioning the objectivity of such information due to perceived conflicts of interest.

*“I consider information from the statutory health insurances very critically. What they would like us to do is very much driven by their own interests.”* (*GP07*,* male*).

##### Industry – DiGA manufacturers/developers

Few GPs expressed a need for more transparent and proactive information from DiGA manufacturers. At the same time, they highlighted the tension between informative content and commercial interests. One GP drew a parallel to pharmaceutical representatives, stressing a preference for neutral sources, excluding advertisement.

##### Journals (independent medical journalists and researchers)

Some GPs expressed a need to receive DiGA-related information through familiar and trusted journals such as the “Arznei-Telegramm” which was valued for its independent, critically reviewed pharmaceutical insights, and considered trustworthy for its lack of commercial bias. Some GPs also recommended the German medical journal “Deutsches Ärzteblatt” as a source for obtaining information about DiGAs.

##### Clinical knowledge platforms

Some physicians identified digital medical educations platforms such as “AMBOSS” and “Deximed” as reliable and familiar sources of information, offering evidence-based content including CME courses. These platforms provide guideline-based information without advertisements but are usually not free of charge (subscription pay model).

##### Quality circles for collegial exchange

Several GPs expressed a preference for exchange within quality circles or informal peer and expert discussions. They also highlighted the value of interdisciplinary dialogue, for example with psychotherapists or psychiatrists.

*“I’ve been moderating quality circles for many years. There’s always collegial exchange in that context. We also discuss case studies. I could imagine doing that for DiGAs as well. Those are useful forums for sharing experiences. Besides that*,* more extensive reports or expert assessments from psychiatrists*,* psychosomatic specialists*,* or psychotherapists would be desirable.”* (*GP10*,* male*).

### Perspectives regarding information content (RQ 5: What type of content do GPs consider most relevant when learning about DiGAs?)

#### Evidence base

A central concern was the need for an easily accessible and transparent presentation of scientific evidence for both temporarily and permanently listed DiGAs, ideally in the form of concise summaries or reviews prepared by trusted institutions or experts. Some GPs emphasized that they do not have time to read individual primary studies but wished for reliable syntheses outlining key measures and outcomes across DiGAs. GPs expressed information wishes regarding controlled studies demonstrating the effectiveness of DiGAs compared to standard treatments or non-DiGA-based interventions. Ideally, such information should elaborate if findings of the DiGA studies are generated based on adequate sample size and methodological sound design, such as including comparisons with first-line treatments.

#### Indications and contraindications

Analogous to conventional drug information, participants emphasized the need for a clear and concise presentation of indications and potential contraindications for DiGAs. Although such information is generally available for each DiGA on the BfArM’s static directory website [53], several GPs reported being unaware of this resource or not consulting it in practice. This may explain why they nevertheless highlighted indications and contraindications as a key information need.

#### Structure, design and usability of DiGAs

Some GPs indicated a need for information on the structure, usability, and design of DiGAs. Particular emphasis was placed on user-friendliness and accessibility for older or less tech-savvy patients, with suggestions that seeing the actual interface and workflow would help assess whether a DiGA is straightforward to use or overly complex.

*“I would appreciate a training that introduces the most commonly used DiGAs*,* including what they look like and how their user interface is designed.” (GP08*,* female).*

Unlike efficacy or effectiveness, most GPs assumed that data security was another criterion for DiGA approval due to the involvement of the BfArM, and they expressed little interest in detailed information.

#### Language availability

Another relevant aspect concerned the availability of DiGAs in multiple languages, such as English, Turkish, or Ukrainian. A few participating GPs highlighted the importance of this criterion for the care of patients with deficits in the German language and multilingual patient groups, for the optimal use of DiGAs.

#### User and peer feedback on practical use

Authentic user experiences were also considered highly valuable. Some GPs reported an interest in obtaining authentic feedback and firsthand experiences from both patients and colleagues regarding the usability and perceived effectiveness of specific DiGAs.

*“For instance*,* I would like to know how many people actually use this app and how patients evaluate it. Such as how many points it gets and whether it is considered helpful. If an app scores only 2 out of 10*,* I wouldn’t prescribe it*,* of course.”* (*GP09*,* male*).

#### Cost and budget information

GPs emphasized the importance of transparent and regularly updated information about DiGA pricing, both to inform patients and to assess possible implications for their own practice budgets.

*“I want to know how much it costs to prescribe a DiGA once. Most of them run for one quarter*,* and ideally*,* I would like to have a table that gives me an overview: how much does the DiGA cost*,* how much time is required for it*,* what is the benefit of it and in how far does it affect the budget – not just the collective healthcare system*,* but also my own budget.”* (*GP11*,* female*).

In particular, GPs wanted to know whether prescribing a DiGA would affect their practice budget or lead to potential financial liabilities, such as regress claims. Specifically, GPs stated being unsure whether DiGA prescriptions are handled outside of regular practice budgets to prevent financial disadvantages. In addition, they called for better integration of cost information into practice management software, similar to how prices of conventional medications are displayed.

### Perspectives regarding delivery modes (RQ 6: Through which delivery modes do GPs want to receive information about DiGAs?)

GPs mentioned diverse formats through which they would like to receive information about DiGAs, ranging from digital, hybrid to in-person options.

#### Online formats

Virtual formats, such as webinars, email newsletters, or online channels on their smartphone, were frequently appreciated for their time-efficiency and flexibility.

*“Ideally*,* I’d like an online news channel that you can access on your phone.”* (*GP07*,* male*).

#### In-person formats

In contrast, others explicitly favored in-person formats, particularly for engaging with new topics such as DiGAs. In this context, a few participants highlighted the value of visually enriched presentations, such as lectures supported by short videos or app screenshots, as helpful for understanding the structure and function of DiGAs. Interactive settings, such as workshops or quality circles, were valued for enabling in-depth discussion, and collegial exchange.

#### Print formats

Some participants emphasized the value of printed materials, such as flyers for quick access to key information, and journals or textbooks for scientific content.

*“I still prefer non-digital media because I can flip through them multiple times and make annotations. I favor journals and textbooks. However*,* books tend to me more static and are updated infrequently*,* so journals are ultimately the more suitable source.” (GP10*,* male).*

### Perspectives regarding time investment (RQ 7: How much time are GPs willing to invest in learning about DiGAs?)

Generally, GPs referred to their very limited time available for each patient, which makes it highly important to establish immediate access to up-to-date information in everyday clinical work (e.g., in the practice software) and stated that they would invest from five to 30 min in daily routine. Nonetheless, as GPs felt not well informed about DiGAs overall, they also indicated a readiness to invest more than one hour in a first in-depth information acquisition. Greater time investment was seen as justified when the practical benefit in everyday professional life was clearly recognizable.

*“If it’s practical training that genuinely supports my daily work*,* I would gladly invest three or four hours. But if it’s only an overview and not directly relevant to practice*,* then I wouldn’t want to invest more than an hour.”* (*GP11*,* female*).

In this context, participants discussed possible incentives to support DiGAs adoption in practice. Several GPs suggested that participation in DiGA-related training sessions should be rewarded with CME credits. Others emphasized the need for financial compensation for the additional effort associated with patient education and prescription.

## Discussion

### Main findings

The present study examined GPs’ perspectives on information needs related to DiGAs for mental health and identified three primary findings. First, GPs reported a relatively high level of engagement with DiGA prescriptions despite persistent knowledge gaps. Second, their attitudes toward digitalization, including DiGAs and AI, were characterized by ambivalence, balancing recognition of potential benefits with concerns about limitations. Third, GPs articulated clear expectations regarding information provision, encompassing trusted sources, relevant content, and suitable delivery formats. These three aspects are discussed in more detail in the following sections.

### Familiarity with and attitudes toward DiGAs

Interestingly, 12 out of 13 participating GPs had prescribed DiGAs at least once, which is notably higher than adoption rates reported in previous studies [22]. Despite this unexpected finding on first prescription experiences, GPs in our study expressed ambivalent views regarding the usefulness of DiGAs. On the one hand, GPs acknowledged advantages such as availability, flexibility, and the potential to bridge waiting times for psychotherapy or mitigate workforce shortages. On the other hand, they raised concerns about administrative burden, lack of technical integration, and possible compromises in the therapeutic relationship, underscoring the continued importance of face-to-face care. This reflects a central tension: While DiGAs have the potential to address gaps in (mental) healthcare, particularly in rural regions, an overemphasis on digitalization could divert attention from the necessity of ensuring a well-funded healthcare workforce [54]. In previous studies physicians suggested that DiGAs should be regarded as complementary rather than substitutive resources [55], and therefore policy strategies must balance investment in digital innovation with measures to strengthen workforce capacity [56].

Furthermore, GPs doubted the usability of DiGAs for older patients, perceiving them as less comfortable with digital technologies. This aligns with findings from a discrete choice experiment (DCE) by Leigh et al. [57] showing reduced prescribing with increasing patient age. Such perceptions may reinforce existing barriers to DiGA adoption among the elderly, although evidence indicates that targeted support can enhance uptake and outcomes in older patients [58].

Attitudes toward AI were also mixed. However, it was generally perceived as a potential tool to reduce workloads. In contrast, prescribing and integrating DiGAs, including the effort of informing oneself about them, was viewed as an additional burden with uncertain benefits for patients and healthcare providers. Consistent with previous research, participating GPs reported very limited knowledge about AI [59], even less than about DiGAs. Similar to earlier findings, some did not see clear clinical use cases for primary care, apart from possible future support in decision-making and diagnosis, whereas they considered applications in other fields, such as radiology, more likely [60]. In summary, DiGAs were primarily discussed in the context of patient care and mental health, while AI was often framed in terms of structural and administrative support. As AI is increasingly being integrated into digital health interventions [41] and DiGAs [40], our findings provide important insights into how GPs perceive and differentiate between such technologies.

Notably, the prescription of DiGAs does not always stem from a clear personal conviction. In several cases, DiGAs were prescribed based on patient requests rather than as part of an active therapeutic decision. While previous studies have emphasized the importance of knowledge in fostering prescribing intentions [21], the present findings suggest that, despite limited knowledge and ambivalent attitudes, actual prescribing may still occur. This suggests a complex interplay between internal readiness and external influences, such as patient-driven demand, which may play a critical role in adoption of DiGAs.

The discrepancy between prescription experience, level of knowledge, and personal attitudes underscores the need for more targeted, accessible, and practice-oriented informational resources to support evidence-based decision making in everyday care. In this context, the present findings highlight the importance of identifying concrete information strategies tailored to the needs of GPs. The qualitative interviews revealed a range of needs and wishes regarding information sources, content, and delivery modes.

### Wishes regarding information sources

GPs emphasized the importance of obtaining information from authoritative and independent institutions within the healthcare system. Central institutions such as BfArM, BMG, and IQWiG were consistently regarded as trustworthy sources [61]. Such centrally endorsed information sources on DiGAs could be more actively or visibly disseminated through channels commonly used by GPs, while also emphasizing the need to increase awareness and utilization of existing resources like the DiGA directory [53]. Optimizing the usability and accessibility of the directory could further facilitate its uptake in everyday practice.

In addition, regional actors such as medical associations and statutory health insurance physicians’ associations were mentioned as generally credible sources of up-to-date information, especially concerning the dynamic field of health policy with changing regulations and reimbursement conditions.

GPs further expressed a need for clear and non-binding guideline-like recommendations to support decision making. Guidelines from professional associations are among the most frequently used sources of information [39, 62, 63]. This underscores the broader relevance of authoritative guidance across specialties. In the context of dMHI, the German guideline for unipolar depression lists such tools as treatment options [64]. However, DiGAs are not explicitly mentioned, despite constituting a specific subset of dMHI that are CE-certified DTx listed in the BfArM registry. This may contribute to the impression that DiGAs are currently not perceived as part of the routine primary care toolkit, particularly in the field of mental health, which some GPs viewed as outside their scope of responsibility.

GPs regarded independent medical journals as reliable and familiar sources of information on DiGAs. In particular, they appreciated journals such as Arznei-Telegramm for their critical stance and transparency regarding commercial interests. Journals can therefore play an important role by providing independent commentary, critical appraisals of evidence, and practical implications tailored to general practice. Previous research has shown that journal articles are among the most frequently used sources of information by physicians [39]. Integrating DiGA-related content into such established publications could enhance credibility and promote informed decision making in clinical practice.

Furthermore, GPs expressed a need to obtain information on DiGAs from clinical knowledge platforms such as AMBOSS and Deximed. These platforms can be considered as point-of-care knowledge resources, as they provide concise, evidence-based, and regularly updated content directly at the site of patient care [65, 66]. Previous research has shown that physicians particularly value such resources for their credibility, efficiency, and seamless integration into clinical workflows [62, 65]. Therefore, providing and extending DiGA-related content coverage on these platforms may enhance GPs’ ability to access pertinent information quickly and efficiently when needed.

Authentic and real-life experiences shared by colleagues were deemed crucial. Reviews support the relevance of colleagues and experts as important information sources for GPs [39, 63]. The interest for interdisciplinary collaboration with other medical disciplines has also been previously reported [63]. Peer exchange, particularly with colleagues encountered in daily professional routines, may serve as a valuable resource to remain informed about medical innovations. In a qualitative study, 57% of insurance physicians stated they expected institutional support for implementing dMHI [32]. However, most GPs in Germany are self-employed (i.e., *n* = 108.200 versus employed: *n* = 60.100 [67]) and do not receive organizational support from higher institutions. Hence, it appears particularly important for self-employed GPs to rely on collegial support within their limited time constraints (e.g., within quality circles).

Additionally, statutory health insurances and DiGA manufacturers were mentioned as information sources. Similar to the findings in a sample of rheumatologists in Germany [68], GPs in our study held divided opinions regarding the trustworthiness of these information sources. While some expressed skepticism and emphasized the role of independent sources, others articulated a need for increased collaboration between these actors with healthcare institutions. These divided attitudes suggest that health insurers and manufacturers may not serve as the most suitable primary sources of DiGA-related information.

### Wishes regarding information content

Regarding information content, GPs emphasized the need for concise, evidence-based data on effectiveness, as well as clearly structured information on indications and contraindications, that can easily be applied to daily practice. Equally important was patient-centered information, including explanations of the structure and usability of DiGAs to support physicians in assessing their suitability for different patients. Several participants also highlighted the need for content available in multiple languages, which would facilitate accessibility and understanding, particularly for non-German-speaking patients. This aligns with findings by Posselt et al. [26] who reported that GPs considered language diversity essential for ensuring equitable access to digital health services.

Notably, GPs highlighted that authentic experience reports from colleagues and patients represent a valuable type of content. Previous studies have shown that perceived effectiveness and efficiency are the most important factors for the professional integration of DTx among HCPs [32, 69, 70]. This may explain why GPs value both empirical and experience-based (narrative) evidence on the usefulness of novel interventions in their clinical decision making.

Consistent with findings by Schroeder et al. [24], who surveyed physicians across medical specialties in Germany, GPs in our study highlighted a need for more detailed information on the financial aspects of prescribing DiGAs, including patient-related costs and potential consequences for their own practice. GPs nevertheless indicated limited familiarity with core aspects such as the evidence base, appropriate indications as well as contraindications, and costs. While such information is generally provided on the DiGA directory since fall 2020, several GPs in our study were either unaware of this resource or did not routinely use it (e.g., due to inconvenient search and filter options, or information overload [71]). Moreover, the structural design of the directory has remained largely unchanged since the introduction of DiGAs, which may further limit its usability, particularly for less experienced users [71]. These gaps and structural shortcomings highlight that mere availability of information does not guarantee its uptake in everyday practice, underscoring the need for more proactive dissemination and integration of DiGA-related content into trusted and frequently used information channels.

### Delivery modes of information

**Fig. 2:**
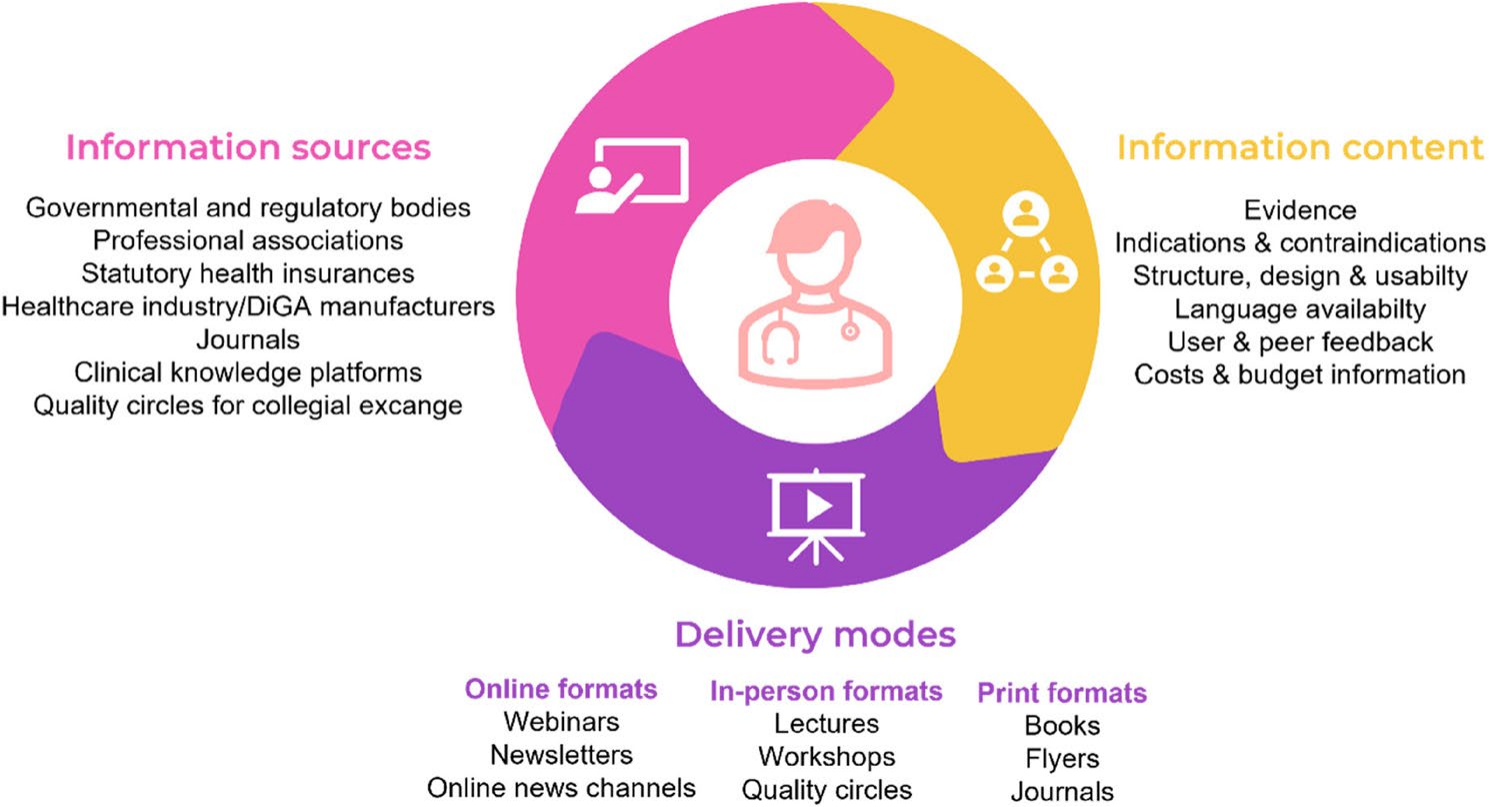
General practitioners’ multifactorial needs for information strategies about digital health applications (DiGAs). Abbreviations: DiGA = Digital health application (German „Digitale Gesundheitsanwendung“). Illustration of GPs’ information needs regarding digital health applications (DiGAs) with the three central components: [1] information sources [2], information content [3], delivery modes

Given their limited time resources, GPs emphasized the need for fast and flexible delivery modes, including online formats and print materials. Notably, participants still placed a high value on traditional text-based formats, such as flyers, (scientific) journals, and books. One reason cited pertains to the capacity to add notes to printed texts, which was perceived as conducive to personal processing. Earlier studies confirm the relevance of such media for GPs, showing that journal articles are seen as current and reliable, while textbooks (even when partially outdated) are still considered trustworthy, relevant, and accessible (see review by Daei et al. [39]). Nevertheless, GPs were also open to attending in-person events related to DiGAs such as lectures and workshops, if a clear benefit was perceived. In particular, workshops were valued for their interactive nature and the opportunity for collegial exchange. Previous studies confirm physicians’ interest in medical conferences and CME events focusing on pharmaceutical updates [72]. Figure 2 illustrates an overview of the GPs multifactorial needs for information strategies about DiGAs.

### Additional factors

The inclination toward efficiency was further reflected by the time participants were willing to allocate to information acquisition, which ranged from five minutes to several hours depending on the format and content. This illustrates that there is no one-size-fits-all approach to information strategies for GPs, as they depend on motivation (including perceived usefulness), prior experience, or knowledge level, as well as structural factors, including limited time, and IT infrastructure. Accordingly, information strategies should consider the limited time resources of GPs in primary care and provide information in formats that can be flexibly accessed over different time spans, enabling GPs to engage with the content according to their schedules.

During several interviews, it became evident that acquiring information regarding DiGAs was not a priority for some GPs. They indicated that other medical topics were more pressing in their daily practice and mentioned information overload as a barrier. In light of the rapidly evolving medical information landscape [30, 31], our findings highlight persistent knowledge gaps regarding DiGAs. Despite their incorporation into the German healthcare system five years ago, DiGAs are still predominantly taught as elective subjects within medical education [73, 74], reflecting the gradual and delayed adaptation of medical curricula to digital innovations in Germany. Evidence from a recent meta-analysis shows that training interventions for HCPs can enhance knowledge, improve self-efficacy, and increase the frequency of DTx prescriptions [75]. In addition, several GPs in our study emphasized that appropriate incentives, such as financial compensation or CME credits for training, could play a crucial role in fostering motivation to engage with DiGAs and to invest the additional effort required for their implementation. Evidence from previous research shows that both monetary incentives and CME credits can significantly increase physicians’ participation in educational activities [76, 77]. Incorporating such incentives into DiGA-related training could support wider adoption and prescription in primary care.

### Strengths, limitations, and implications

This study provides novel insights into the information needs of GPs regarding DiGAs in the context of digital mental health. A particular strength is that most participants had already prescribed DiGAs, enabling practice-oriented reflections on facilitators and barriers. Their real-world experience allowed for a nuanced discussion of both structural and content-related information gaps. Furthermore, the age distribution (*M* = 49 years) was close to the national average of German GPs in 2024 (*M* = 54.1 years), ensuring input from physicians at different career stages [78].

At the same time, several limitations must be acknowledged. The unusually high proportion of GPs with prior prescribing experience suggests a potential self-selection bias toward digitally interested physicians, with the perspectives of less experienced or more skeptical GPs being underrepresented. In addition, the sample showed an imbalanced gender distribution (10 male, 3 female), which may have biased the range of perspectives represented.

Moreover, the regional concentration in North Rhine-Westphalia, Germany’s most populous state [79], means that views from underserved regions with lower physician density were not adequately captured. This could have biased findings toward better-resourced settings. Future research should purposively include GPs with little or no prescribing experience, as well as a broader regional and gender distribution, to better capture additional barriers such as lack of knowledge, structural constraints, or concerns about patient acceptance.

Another limitation concerns scope. Although the interview guide explicitly focused on DiGAs in mental health, participants often generalized to DiGAs overall. Interviewers attempted to refocus, but responses still extended beyond mental health. While this broader perspective provides valuable insights, specific conclusions about mental health DiGAs should be interpreted with caution.

Despite these limitations, the study highlights the heterogeneity of GPs’ information needs across sources, content, and delivery modes, suggesting that a “one-size-fits-all” strategy is unlikely to succeed. Methodologically, DCEs could help quantify preferences and guide tailored information strategies [80]. Experimental approaches such as randomized controlled trials may further identify effective formats for communicating prescription procedures, evidence, and costs.

From an implementation perspective, findings point to the need for structural and educational adaptations. CME programs and medical curricula should integrate DiGA-related content to build digital competencies early on and reduce knowledge gaps [74]. Future research should examine whether early curricular exposure translates into greater willingness and prescribing frequency. Such studies could provide empirical evidence on the potential benefits of early exposure in reducing knowledge gaps and facilitating adoption in practice. This may not only be applicable to DiGAs but also to subjects such as AI in medical practice. Differences between GPs and specialists should be explored to design more profession-specific training materials.

Finally, given the close link between DiGAs and broader innovations, future studies should also investigate physicians’ attitudes toward AI. Understanding both individual-level (provider–patient) and systemic (infrastructure, regulation) factors shaping acceptance will be crucial for developing strategies that support HCPs in navigating digital transformation.

## Conclusions

The digital transformation of healthcare poses significant challenges for medical practices. To adequately prepare physicians, promoting digital competencies and providing tailored information must become integral components of CME. It is therefore the responsibility of the medical profession to develop digital health training that is forward-looking and responsive to the specific needs and challenges faced by physicians prescribing DiGAs.

## Supplementary Information

Supplementary Material 1

## Data Availability

Anonymized interview transcripts generated and analyzed during the current study are available from the corresponding author upon reasonable request.
