# Supplementary material for: Genie in the bottle? a qualitative study of general practitioners’ perspectives and information needs concerning digital mental health applications in Germany
Source: BMC Prim Care. 2025 Dec 9;26:393. doi: 10.1186/s12875-025-03115-2 (PMC12687503; doi:10.1186/s12875-025-03115-2)
Supplement: Supplementary file 1 — Supplementary Material 1 [file 12875_2025_3115_MOESM1_ESM.docx]

Supplementary Material

Supplementary Table 1. COREQ Checklist.

| No. | Item | Description | Section/Notes |
| --- | --- | --- | --- |
| Domain 1: Research team and reflexivity | | | |
| Personal characteristics | | | |
| 1 | Interviewer/ facilitator | Which author/s conducted the interview or focus group? | See section Research Team |
| 2 | Credentials | What were the researcher’s credentials? *E.g. PhD, MD* | See section Research Team |
| 3 | Occupation | What was their occupation at the time of the study? | See section Research Team |
| 4 | Gender | Was the researcher male or female? | See section Research Team |
| 5 | Experience and training | What experience or training did the researcher(s) have? | See section Research Team |
| Relationship with participants | | | |
| 6. | Relationship  established | Was a relationship established prior to study commencement? | See section Sampling Strategy |
| 7. | Participant knowledge of the interviewer | What did the participants know about the researcher? *E.g. personal goals, reasons for doing the research* | See section Sampling Strategy and Study Design |
| 8. | Interviewer  characteristics | What characteristics were reported about the interviewer/facilitator? *e.g. Bias, assumptions, reasons and interests in the research topic* | See section Study Design, Research Team |
| Domain 2: Study design | | | |
| Theoretical framework | | | |
| 9. | Methodological  orientation and  Theory | What methodological orientation was stated to underpin the study? *E.g. grounded theory, discourse analysis, ethnography, phenomenology, content analysis* | See section Methods and Study Design |
| Participant selection | | | |
| 10. | Sampling | How were participants selected? *E.g. purposive, convenience, consecutive, snowball* | See section Sampling Strategy |
| 11. | Method of approach | How were participants approached? *E.g. face-to-face, telephone, mail, email* | See section Sampling Strategy |
| 12. | Sample size | How many participants were in the study? | See section Sampling Strategy, Sample characteristics |
| 13. | Non-participation | How many people refused to participate or dropped out? What were the reasons for this? | See section Sampling strategy |
| Setting | | | |
| 14. | Settings of data collection | Where was the data collected? *E.g. home, clinic, workplace* | See Section Study Design |
| 15. | Presence of non-participants | Was anyone else present besides the participants and researchers? | See Section Study Design |
| 16. | Description of sample | What are the important characteristics of the sample? *E.g. demographic data, date* | See section Sample characteristics, Table 1 |
| Data collection | | | |
| 17. | Interview guide | Were questions, prompts, guides provided by the authors? Was it pilot tested? | See Section Study Design and Supplementary Table 3 |
| 18. | Repeat interviews | Were repeat interviews carried out? If yes, how many? | No |
| 19. | Audio/visual recording | Did the research use audio or visual recording to collect the data? | See section Study Design |
| 20. | Field notes | Were field notes made during and/or after the interview or focus group? | See section Study Design |
| 21. | Duration | What was the duration of the interviews or focus group? | See section Results |
| 22. | Data saturation | Was data saturation discussed? | See section Sampling Strategy |
| 23. | Transcripts returned | Were transcripts returned to participants for comment and/or correction? | No |
| Domain 3: Analysis and findings | | | |
| Data analysis | | | |
| 24. | Number of data coders | How many data coders coded the data? | See section Research team and Data analysis |
| 25. | Description of the coding tree | Did authors provide a description of the coding tree? | See section Data analysis and Supplementary Table 4 |
| 26. | Derivation of themes | Were themes identified in advance or derived from the data? | See section Data analysis |
| 27. | Software | What software, if applicable, was used to manage the data? | See section Data analysis |
| 28. | Participant checking | Did participants provide feedback on the findings? | No |
| Reporting | | | |
| 29. | Quotations presented | Were participant quotations presented to illustrate the themes/findings? Was each quotation identified? *E.g. Participant number* | See section Results |
| 30. | Data and findings consistent | Was there consistency between the data presented and the findings? | See section Results |
| 31. | Clarity of major themes | Were major themes clearly presented in the findings? | See section Results |
| 32. | Clarity of minor themes | Is there a description of diverse cases or discussion of minor themes? | See section Results |

Supplementary Table 2. Brief background questionnaire (translated, German to English).

| **Pseudonymization code** (to be filled in by the investigator)**:** _________________ |
| --- |
| **Personal details** |
| (1) How old are you at the time of the scheduled interview? ______________ years |
| (2) What is your gender?  ☐ female ☐ male ☐ diverse/other |
| (3) Which degree program/s have you completed?  (multiple answers are possible)  ☐ Human medicine ☐ Psychology  ☐ Other, namely: __________________________________ |
| (4) Have you completed specialist medical and/or psychotherapeutic training?  ☐ Yes, in the specialist field: ____________________________________  ☐ No, I am currently not in (further) training  ☐ No, but I am currently in further training,  in the subject/subject area: ____________________________________ |
| (5) In which federal state do you work as a specialist? _______________________________ |
| (6) How do you rate the location of your place of work?  ☐ (rather) rural ☐ (rather) urban |
| (7) To what extent are you currently working?  ☐ Yes, full-time,  ☐ Yes, part-time,  ☐ Yes, other, namely _____________________________  ☐ No, comment (optional): _____________________  ☐ No specification |
| (8) If you are self-employed: Do you have a statutory health insurance license?  ☐ Yes ☐ No |
| (9) How many years of practice do you have? ______________ years (since graduation) |
| **Awareness of digital interventions for mental health purposes** |
| (10) Have you heard of specific digital health interventions for mental health (digital Mental Health Interventions, dMHI) for preventive purposes (e.g., stress reduction and/or treatment included before participating in the study?  ☐ No  ☐ Yes, namely in the area/s, optional answer: __________________________________________  ________________________________________________________________________________ |
| (11) If you answered “yes” to question 10:  Have you ever prescribed a digital health intervention (German: Digitale Gesundheitsanwendungen, DiGA)?  ☐ No  ☐ Yes, namely (e.g., name of the DiGA or indication/diagnostic group):  _______________________________________________________________________________ |
| (12) Are you already advising patients resp. clients on DiGA or DMHIs?  ☐ Yes, I have already given advice on this  ☐ I am still uncertain (see question 13)  ☐ No, not interested (commentary, optional): _____________________________________ |
| (13) Do you intend to advise your patients resp. clients on DiGA or DMHIs in the future?  ☐ Yes, it is planned  ☐ Yes, but I don’t feel adequately prepared, yet (commentary, optional):  ________________________________________________________________________________ |

Commentary (optional):

_______________________________________________________________________________

_______________________________________________________________________________

_______________________________________________________________________________

_______________________________________________________________________________

Supplementary Table 3. Guideline for semi-structured interview (translated, German to English)

*** = Optional follow-up questions, e.g., if the conversation stalls

| **Procedure/topics** | **Suggested questions** |
| --- | --- |
| **Demands and needs** | |
| **0 Introduction & presentation** | Introduction of interviewer, study objectives („*Determine information preferences to maybe design optimal strategies for professionals*“), procedure explanation (at the start of the interview: *Video off, start of recording, please do not provide any personal data, etc.* – end: *recording will be stopped, video on*), clarification of open questions  Brief explanation of the terms „*digital Mental Health Intervention (dMHI)*: *All interventions that support health electronically … this includes so-called digital health applications (short: DiGA – “apps on prescription”)* |
| **1 Possible introductory question(s)**  (personal questions in the questionnaire), survey of attitudes | How did you hear about our study? *(Note: avoid clearly assignable information, otherwise blacken).*  What do you associate with the term dMHI/DiGAs? What chances or risks do you see (for your specialization, for patients (P)/clients (C))?  *** What is your general attitude towards the issue of prescribing DiGAs or use of dMHI as a general practitioner (GP)/psychotherapist (PT)?  *** What opportunities and risks do you associate with dMHI/DiGAs? |
| **2 Possible (follow-up) questions to the topics of *prior knowledge & familiarity with the topic***  (if necessary: Specific questions on the brief pre-interview questionnaire) | What do you already know about dMHI/DiGAs?  How well informed do you feel about dMHI/DiGAs, e.g., to advise P/C? Where do you think is room for improvement?  *** Which dMHI or DiGAs for mental health do you know (e.g., free available or prescribable apps)?  (*** How familiar are you with this?)  *** Do you know special DiGAs (“apps on prescription“)? What do you think of DiGAs? Have you ever prescribed a DiGA? (If so: which one? If no: why not?; alternatively: What are arguments for or against prescribing DiGAs?) |
| **3 Possible (follow-up) questions to the topics of *digital health in the professional career and environment*** | (To what extent) Have you already received/discussed information (prescription, type of services, etc.) on dMHI/DiGAs (reminder: digital mental health interventions resp. digital health applications) in your professional career (studies, training, further education)?  To what extent has the topic of dMHI/DiGAs been dealt with in your working environment (e.g., clinic, internal training, etc.)?  *** How do your superiors and/or colleagues think about this? To what extent is there an exchange on the topic and/or on existing trainings to dMHI/DiGAs?  *** What do you think about receiving information about dMHI/DiGAs from other professionals or exchanging information (e.g., mentors, team meetings)?  What benefits/incentives would motivate you to take a closer look at dMHI/DiGAs? |
| **4 Possible (follow-up) questions to the topics of *experience with use (patients), general wishes/preferences for use*** | Can you estimate the time required to prescribe a DiGA?  Is a prescription realistic in your work routine?  *** What would you need (additional material, etc.) to ease the process?  What would be your motivation for prescribing a DiGA?  Which offers could be particularly relevant for your P/C?  For which indication would you prescribe a DiGA (reminder: app on prescription)? Would you exclude or favor certain P/C or age groups?  Do you know whether your P/C already used dMHI/DiGAs?  *** If you have already received feedback from P/C according to the use of dMHI/DiGAs: What may be facilitating or hindering factors for the use?  *** If you have already recommended or prescribed DiGAs: What experiences did your P/C report? What is the general resonance?  **** How do you experience cooperation with other authorities concerning DiGAs, e.g., cooperation with providers, health insurance companies, P/C? |
| **Wishes (preferences)** | |
| **5 Possible (follow-up) questions to the topics of *wishes/preferences concerning the search for (online) health information, and presentation modalities***  Time and effort | How do you proceed to obtain information on new health services (especially in the field of mental health)?  As a GP or PT, in which way/through which medium would you like to be informed about existing offers?  Which media (virtual vs. non-virtual) do you prefer here?  ***Which media/information channels do you prefer to obtain relevant information quickly?  How much time are you willing to invest in the (first) search, for a first impression, for information on new offers or to check offers? (*note: this is about the first information/impression*)  How much are you willing to invest in further training on dMHI/DiGAs (time, effort, other costs)? |
| **6 Possible (follow-up) questions to the topics of *wishes/preferences for the content of information on dMHI/DiGAs***  (quality criteria, important attributes) | What information (in terms of content) is important to you, to make an informed decision about recommending or prescribing a DiGA?   - What would you like to know in advance? What influences your decision? (What quality features come to your mind?) - Which types of dMHI/DiGAs, for which indication, would be particularly helpful for your P/C and therefore important to know?   What information would you prevent/hinder recommending/prescribing a DiGA?  *** What role does data protection information play for you?  How important is information on costs or cost coverage (for advising your P/C)? How detailed do you wish this information to be?  *** What are the three most important contents of information materials from your point of view? |
| **7 Possible (follow-up) questions to the topics of *design and* *presentation* *wishes/preferences for information on* dMHI*/DiGAs*** | What appeals to you in terms of design if you were about to inform about dMHI/DiGAs?  In what form should the information be prepared or communicated (e.g., text, videos, podcasts, texts with images, etc.; different media formats)?   - What scope ([text] length or video/podcast [duration]) do you prefer? (time, effort depending on media) - How important is interactivity to you when acquiring information (e.g., workshop vs. lecture)   *** What do you not like? (e.g., something like visually overwhelming, overloaded websites, too much technical language)  How should the information be presented linguistically/formally? (if you want to inform yourself and if you want to refer P/C to information material) |
| **8 Possible (follow-up) questions to the topics of *trustworthiness, credibility & recommendations*** | What source of information do you find trustworthy? Who is important to you as a source of information? (*on request, if there is no answer: examples such as health insurance companies, journals, university, media, colleagues, etc.)*  What exactly creates trust for you/makes information on apps credible? (maybe again asking whether DiGA directory is known)  *** How do you feel about quality seals/seals of approval/certifications for apps? Which ones do you know?  What do you think of testimonials (concerning information material)? Who should inform P/C? (e.g., GP/PT/other experts, other P/C …)  How important is information on scientific evidence to you, and how detailed should it be?  Who should provide the information (for professionals)? (e.g. [medical] associations, research institutions, department of health, etc.) |
| **Closing** | |
| **9 Possible (follow-up) questions to the topics of**  ***Further wishes and suggestions*** *(open)*  ***“Mircale question”***  *Additional, starting July 27, 2024:* ***Attitudes toward artificial intelligence (AI)***  **Farewell** | Do you have any suggestions or are there any aspects that we have not yet discussed?  *** What would be the optimal dMHI/DiGAs training for you as a professional (GP/PT), for example, if you had unlimited resources (in relation to requests for information strategies)?  *** What would be the optimal dMHI/DiGAs offer for your P/C or special P/C groups, if you had unlimited resources (in relation to intervention requests)?  How important do you think it is for medical professionals to be informed about the latest AI developments?  Do you have any specific requests or ideas about how you would like to be informed most effectively about new developments in the field of AI?  End of interview, thanks for participating, clarify open questions, information to process of the expense of compensation |

*Note.* The guideline may change during the survey. This is common in qualitative research to react to unexpected content. Abbreviations: AI = Artificial intelligence, C = Client, DiGAs = Digital health applications (German *Digitale Gesundheitsanwendungen*), dMHI = Digital Mental Health Interventions, GP = General practitioner, P = Patient, PT = Psychotherapist.

Supplementary Table 4. Excerpt from the coding scheme and anchor examples (translated, German to English)

| **Level 1** | **Level 2 (Level 3)** | **Anchor examples** |
| --- | --- | --- |
| **RQ 1: How do GPs perceive their current level of knowledge regarding DiGAs?** | | |
| Current state of knowledge ^d^ | High level of knowledge ^i^ | *“There are, of course, those very specifically, predominantly behavior therapy-oriented apps that do quite a lot themselves, interacting with the patients beyond psychoeducation. And this becomes relevant when patients are familiar with the electronic devices required and when they’re willing to use them. And when the app is at least on the official list of the Federal Institute for Drugs and Medical Devices. There are different stages of approval, and for me it’s important that they are permanently listed products.”* (*GP13, male*). |
|  | Middle level of knowledge ^i^ | *“I know DiGAs are electronic applications that patients can download, and that they are a type of app. I also know that there must be a formal approval process for them, which determines whether the statutory health insurance covers the cost. I am aware that they need to undergo a certain certification procedure and fulfill specific criteria in order to be eligible for reimbursement.” (GP07, male)* |
|  | Low level of knowledge ^i^ | *“I know there are applications that allow people to log in online and access certain services. But to be honest, I’m not exactly sure how it all works. Apparently they receive some form of online consultation.” (GP12, male)* |
| **RQ 2: What are GPs' attitudes toward digitalization in medical practice, including the diffusion of DiGA and Artificial Intelligence (AI) into primary care?** | | |
| Attitudes toward digitalization in medical practice ^d^ | Attitudes toward DiGAs (level 3: positive; indecisive; negative) ^i^ | *“Especially in the field of mental health, I consider DiGAs very useful. Many patients have urgent needs, but there is just a lack of human or physical capacity to take care of it. As general practitioners we are also bound by strict regulations and time constraints in what we can offer. DiGAs can be used by patients at any time. That’s why I would say I’m generally positive toward it.”* *(GP01, male).* |
|  |  | *„In everyday practice, DiGAs have so far played only a minor role. I’m not quite sure why that is.“* (*GP06, male*). |
|  |  | *“What makes DiGA unattractive to me is the fact that it shifts everything to an impersonal, ultimately AI-based level, where personal exchange, direct communication, and the emotional dimension between people no longer take place.”* (*GP07, male*). |
|  | Attitudes toward AI (level 3: positive; indecisive; negative) ^i^ | *“I am very much in favor of AI, particularly when it helps reduce the workload. This includes diagnostic support, of course, but also administrative tasks such as billing. Any form of relief that does not require additional staff would be highly appreciated.”* (*GP11, female*). |
|  |  | *“At the moment, I don’t see any advantage of AI, except maybe for radiology findings, standard findings.”* (*GP04, male*). |
|  |  | *“I would say that I am basically also rather critical toward AI and I have quite some respect for the development and the use of these technologies, so I don’t think that keeping medical staff informed about every latest innovation in this field should be a top priority.”* (*GP07, male*). |
| **RQ 3: Whose initiative leads to the prescription of DiGAs in general practice?** | | |
| Initiators of DiGA prescription ^i^ | GP initiative ^i^ | *Occasionally, we actively prescribe DiGAs by directly suggesting them to patients, especially in cases involving mental health issues. Since it often takes quite a while for patients to access psychotherapy, DiGAs can serve as a useful interim solution until a therapy slot becomes available.” (GP09, male)* |
|  | Patient initiative ^i^ | *“Occasionally, I have patients, who explicitly request such applications. In those cases, I issue the prescription. I was fine with that.”* *(GP12, male)*.  *“I received several faxes from a DiGA provider, including the name of my patients and asking me to return the completed form or prescription. I think that is presumptuous and intrusive.” (GP06, male)* |
| **RQ 4: Which information sources do GPs consult when seeking information about DiGAs?** | | |
| Perspectives regarding information sources ^d^ | Governmental and regulatory bodies ^i^ | *“For me, the BfArM would be the number one trusted source for information on DiGAs.” (GP09, male)*  *“I think that, if anything, information should come from the Association of Statutory Health Insurance Physicians, since they decide what can be prescribed. The information should be centralized, I don’t care whether this is through the medical associations or the regional insurance physicians’ associations, but ideally, through a separate website.” (GP02, male)*  *“In the field of occupational medicine, I fully trust the information provided by the Federal Institute for Occupational Safety and Health. I know that the content is reviewed up-to-date, and reliable.”* (*GP01, male*). |
|  | Professional associations ^i^ | *“In my view, institutions such as DEGAM or the German Association for General Practitioners are trustworthy. In my opinion, they should be the ones providing information about DiGAs.”* (*GP07, male*). |
|  | - 1. Statutory health insurances ^i^ | *"* *I consider information from the statutory health insurances very critically. What they would like us to do is very much driven by their own interests."* (*GP07, male*). |
|  | - 1. Industry – DiGA manufacturers/ developers ^i^ | *“I think DiGA manufacturers could do a bit more. They should bring us on board and explain what different DiGA are available. There is definitely room for improvement.”* (*GP09, male*).  *“I really want to hear this from a neutral source. Whoever offers these programs clearly has a vested interest, of course. It's just like medication. I have not engaged with pharmaceutical representatives in my practice for decades because what they provide is advertising, and I prefer to get neutral information.”* (*GP10, male*). |
|  | Journals ^i^ | *“I would most appreciate information sources like the ‘Arznei-Telegramm, which is entirely independent, non-pharmaceutical journal. It takes a critical look at new medications, sometimes quite scathingly. If a neutral journal like that were to deal with DiGAs, I would find that very helpful. If someone who knows their way around studies and practical experiences could summarize the findings in a compact and structured way, especially tailored for primary care, that would be particularly helpful.”* (*GP10, male*). |
|  | Clinical knowledge platforms ^i^ | *“I would like a reliable source offering a concise overview. I tend to trust established educational platforms such as ‘Amboss’ or ‘Deximed’.” (GP09, female)* |
|  | - 1. Quality circles for collegial exchange ^i^ | *“I’ve been moderating quality circles for many years. There’s always collegial exchange in that context. We also discuss case studies. I could imagine doing that for DiGAs as well. Those are useful forums for sharing experiences. Besides that, more extensive reports or expert assessments from psychiatrists, psychosomatic specialists, or psychotherapists would be desirable.”* (*GP10, male*). |
| **RQ 5: What type of content do GPs consider most relevant when learning about DiGAs?** | | |
| Perspectives regarding information content ^d^ | Evidence base ^i^ | *“In the best case, studies that have shown effectiveness compared to standard treatments or non-DiGA-based digital interventions, based on a controlled study with a sufficient number of participants, would be optimal.”* (*GP11, female*). |
|  | - 1. Indications and contraindications ^i^ | *“Let me draw a parallel to medication prescriptions. For each drug, there is professional information covering its use. We don’t necessarily need dosing information here, but we do need to know exactly which indication is being addressed.”* (*GP10, male*). |
|  | - 1. Structure, design and usability of DiGAs ^i^ | *“I would appreciate a training that introduces the most commonly used DiGAs, including what they look like and how their user interface is designed.”* (*GP08, female*). |
|  | - 1. Language availability ^i^ | *“We have many patients with migration background, for example Turkish-speaking patients who do not speak German well. At the moment, there are also many Ukrainian patients. It would be worth considering whether DiGAs should be offered in languages that are commonly spoken in Germany for those patients. (GP10, male)* |
|  | - 1. User and peer feedback on practical use ^i^ | *“For instance, I would like to know how many people actually use this app and how patients evaluate it. Such as how many points it gets and whether it is considered helpful. If an app scores only 2 out of 10, I wouldn’t prescribe it, of course.”* (*GP09, male*). |
|  | - 1. Cost and budget information ^i^ | *“I want to know how much it costs to prescribe a DiGA once. Most of them run for one quarter, and ideally, I would like to have a table that gives me an overview: how much does the DiGA cost, how much time is required for it, what is the benefit of it and in how far does it affect the budget – not just the collective healthcare system, but also my own budget.”* (*GP11, female*).  *“Ideally these applications should be eligible for budget-neutral prescribing, so that their use does not place financial burden on physicians.” (GP10, male)* |
| **RQ6: Through which delivery modes do GPs want to receive information about DiGAs?** | | |
| Delivery modes ^d^ | Online formats ^i^ | *“Online formats have the advantage of being independent of time and place, so you don’t have to complete them at a specific moment but rather when it fits into your schedule. That’s why I find online formats more practical.” (GP01, male)*  *“Ideally, I’d like an online news channel that you can access on your phone.” (GP07, male)* |
|  | In-person formats ^i^ | *„New topics such as DiGAs need to be addressed in in-person events, because the opportunity for exchange is essential.” (GP04, male)* |
|  | Print formats ^i^ | *“I still prefer non-digital media because I can flip through them multiple times and make annotations. I favor journals and textbooks. However, books tend to me more static and are updated infrequently, so journals are ultimately the more suitable source.” (GP10, male).* |
| **RQ 7: How much time are GPs willing to invest in learning about DiGAs?** | | |
| Perspectives regarding time investment ^d^ | Willingness to invest time ^d^ | *"If it’s practical training that genuinely supports my daily work, I would gladly invest three or four hours. But if it's only an overview and not directly relevant to practice, then I wouldn’t want to invest more than an hour."* (*GP11, female*). |
| Incentives for information gathering ^d^ | DiGA-related training (i.e., CME-events) ^i^ | *“It would be beneficial if participation in information events on DiGAs were accredited with CME points and if these events were more widely promoted.” (GP09, male)* |
|  | Monetary incentives (level 3: financial compensation; extrabudgetary DiGA-prescriptions) | *“If prescribing a DiGA were compensated, for example with 100 euros, the incentive to prescribe would certainly be much higher. That would also be justified, as the process involves a genuine counseling effort. Financial incentives need to be created in order to significantly improve the current situation.” (GP04, male)* |

*Note.* ^d^ deductively coded; ^i^ inductively coded; level 3 codes are presented in parts. *Abbreviations*. AI = Artificial Intelligence; BfArM = German Federal Institute for Drugs and Medical Devices (German *Bundesinstitut für Arzneimittel und Medizinprodukte)*; CME = Continuing Medical Education; DiGAs = digital health applications (German *Digitale Gesundheitsanwendungen*); GP = General Practitioner; RQ = research questions.
